# Neurosurgeon-Neurophysiologist mutualistic symbiosis

**DOI:** 10.1016/j.bas.2024.102750

**Published:** 2024-01-22

**Authors:** José Pedro Lavrador, Ana Mirallave-Pescador, Francesco Vergani

**Affiliations:** Neurosurgery Department, King's College Hospital Foundation Trust, London, UK; Neurosurgery Department, King's College Hospital Foundation Trust, London, UK; Clinical Neurophysiology Department, King's College Hospital Foundation Trust, London, UK; Neurosurgery Department, King's College Hospital Foundation Trust, London, UK

Dear Editor

*Symbiosis* is defined as a close relationship between subjects of different species, where both survive and live together (Paracer and Ahmadjian, 2000). Neurosurgeons and Clinical Neurophysiologists benefit from a similar relationship that occurs when both doctors work together for the benefit of their patient.

Neurosurgery involves significant risks for patients' quality of life and overall survival. Nevertheless, when the treatment goals are achieved, successful treatment can benefit patients. Increased survival despite deprived quality of life from new onset neurological deficits has been rejected by patients as an acceptable treatment outcome.

In the last decades, the neurosurgical community has witnessed a remarkable development in Clinical Neurophysiology and intraoperative neuromonitoring (Skinner and Sala, 2017). New ways to map and monitor specific neurological functions were needed to address lesions in eloquent areas (Holdefer and Skinner, 2017). Intraoperative neuromonitoring expansion has informed the neurosurgical practice.

Assuming appropriate inherent knowledge to each medical and surgical specialty, Neurosurgery and Clinical Neurophysiology relationship implies specific technical and non-technical skills that are applied during the communication in surgical environment.

It is crucial to understand the interventional cascade in both specialities: the Neurosurgeon depends upon communication to be aware about the outcome of their actions whilst the Clinical Neurophysiologist interprets the outcome after the intervention to communicate to the surgeon after being informed about the intraoperative findings (Holdefer and Skinner, 2017). Technical and non-technical competencies from Neurosurgeon and Clinical Neurophysiologist cannot be individualized as they are expressed in *continuum* in this relation – technical competencies: intervention and interpretation; non-technical competencies: information and communication, respectively.

On the one hand, the neurosurgical practice is informed and refined by the additional insight offered by Neurophysiology, while the clinical neurophysiologist can have a better insight into the challenges posed by particular surgical approaches or techniques. A clear and open communication between the two teams is paramount to the good outcome of surgery. In this scenario, the surgeon relies on continuous and live update from the neurophysiology team in order to adjust/correct the surgery, while the clinical neurophysiologist evaluates the outcome and impact of the changes made. Again, technical (i.e. intervention and interpretation) and non-technical (i.e. communication) competencies play a major role and are expressed as a continuum between neurophysiology and neurosurgery.

From both sides, there is a clear perception of an unmet need to improve these technical and non-technical competencies during each speciality training (Holdefer and Skinner, 2017; Ounounou et al., 2019; Hénaux et al., 2019). Particularly on the non-technical competencies, the literature suggest not enough focus on this matter during training despite a general perception about its relevance (Ounounou et al., 2019; Hénaux et al., 2019). We believe that these matters are of high importance and this relationship should be nurtured. A deep understanding of each other develops a relationship of trust, which is both beneficial for work ethics, and the ultimate clinical outcome for patients.

When left unaddressed, a poor communication in theatre paves the way to inadequate treatment outcomes for patients, which can be detrimental to their quality of life and a source of morbidity/mortality. Secondly, this can lead to litigation. Multiple factors contribute to the above: poor communication of warning signs and inadequate techniques of monitoring agreed by both neurosurgeon and clinical neurophysiologist, lack of intervention by the surgical team in the event of neurophysiological warning and poor understanding about the pathology and anatomy that hampers the interpretation of the intraoperative neuromonitoring and mapping (Brook and Irle, 2016).

Therefore, we believe in an *integrated framework* (Fig. 1) where both Neurosurgeon and Clinical Neurophysiologists work together across the different stages of the surgical neurological treatment. Both parts must take part in the *preoperative* neurological assessment and functional mapping of the patients undergoing treatment. This will potentiate an understanding about the risk involved in the procedure and establish a joint treatment plan that will facilitate the intraoperative interpretation of the mapping and monitoring as well as the potential interventions required. *Intraoperatively*, none of the parts should be blinded to their counterpart so that a better information, intervention and communication can be performed. Therefore, the current technological advances allow for imaging integration and/or overlay in the operative microscopes or intraoperative neurophysiology softwares. This provides continuous non-verbal information and communication between both parts. *Postoperatively*, a joint assessment of the patient in recovery and wards and the integration of the intraoperative findings is crucial to ascertain the success of the treatment strategy, to establish a follow-up and/or rehabilitation plan if required (presence of a neurological deficit), and improve the meta-knowledge of the treating team and impact the treatment of the next patient. When outcomes are not as expected, both specialists take responsibility together of solving these complications or providing duty of candour to the patient. Our experience is that patient trust is enhanced when both professionals are part of their clinical care.

**Fig. 1:**
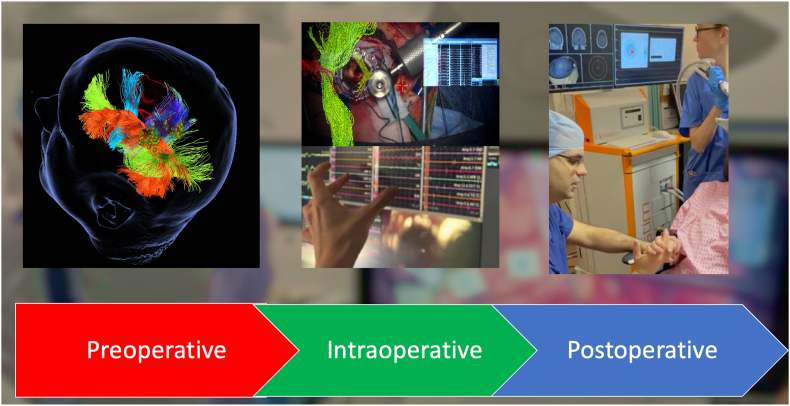
Peri-Operative Neurosurgery-Neurophysiology Integrated Framework – Left: Joint preoperative cortical and subcortical mapping; Centre – Intraoperative share decision making through bilateral integration of IONM and microsurgical information; Right – Postoperative rehabilitation through navigated transcranial magnetic stimulation of a patient with new postoperative motor deficit.

In conclusion, close cooperation between Neurosurgeon and Clinical Neurophysiologist requires both technical and non-technical competencies applied during patient's treatment. An integrated framework where both plan, execute and follow-up the surgical treatment of a neurological patient is the cornerstone for a successful outcome.

## Declaration of competing interest

The authors declare that they have no known competing financial interests or personal relationships that could have appeared to influence the work reported in this paper.

## References

[bib1] Brook M., Irle K. (2016). Litigating intraoperative neuromonitoring (IOM). Univ. Baltim. Law Rev..

[bib2] Hénaux P.L., Jannin P., Riffaud L. (2019). Nontechnical skills in neurosurgery: a systematic review of the literature. World Neurosurg..

[bib3] Holdefer R.N., Skinner S.A. (2017). Commentary : the value of intraoperative neurophysiological monitoring: evidence, equipoise and outcomes. J. Clin. Monit. Comput..

[bib4] Ounounou E., Aydin A., Brunckhorst O., Khan M.S., Dasgupta P., Ahmed K. (2019). Nontechnical skills in surgery: a systematic review of current training modalities. J. Surg. Educ..

[bib5] Paracer Surindar, Ahmadjian Vernon (2000).

[bib6] Skinner S., Sala F. (2017). Communication and collaboration in spine neuromonitoring: time to expect more, a lot more, from the neurophysiologists. J. Neurosurg. Spine.

